# Implementierung einer psychotherapeutischen Sprechstunde am Arbeitsplatz (PT-A): Erwartungen, Bekanntmachung und Nutzung: Ergebnisse der friaa-Studie

**DOI:** 10.1007/s00103-024-03909-2

**Published:** 2024-06-19

**Authors:** Fiona Kohl, Ute B. Schröder, Ralf Stegmann, Uta Wegewitz, Nicole Hander, Marieke Hansmann, Peter Angerer, Yesim Erim, Sinja Hondong, Christoph Kröger, Nadine Mulfinger, Tamara Waldman, Kristin Herrmann, Jeannette Weber

**Affiliations:** 1 Institut für Arbeits‑, Sozial- und Umweltmedizin, Centre for Health and Society, Medizinische Fakultät und Universitätsklinikum Düsseldorf, Heinrich-Heine-Universität Düsseldorf, Düsseldorf, Deutschland; 2 FG 3.5 „Evidenzbasierte Arbeitsmedizin, Betriebliches Gesundheitsmanagement“, BAuA – Bundesanstalt für Arbeitsschutz und Arbeitsmedizin, Berlin, Deutschland; 3 Klinik für Psychosomatische Medizin und Psychotherapie, Universitätsklinikum Ulm, Ulm, Deutschland; 4 Abteilung Klinische Psychologie und Psychotherapie, Institut für Psychologie, Universität Hildesheim, Hildesheim, Deutschland; 5 Psychosomatische und Psychotherapeutische Abteilung, Universitätsklinikum Erlangen, Friedrich-Alexander-Universität Erlangen-Nürnberg (FAU), Erlangen, Deutschland; 6 Klinik für Psychiatrie und Psychotherapie II, Bezirkskrankenhaus Günzburg, Universität Ulm, Günzburg, Deutschland

**Keywords:** Psychische Gesundheit, Früherkennung, Prävention, Psychosomatische Sprechstunde im Betrieb, Betriebliche Gesundheitsförderung, Mental health, Early diagnosis, Prevention, Psychosomatic consultation at work, Workplace health promotion

## Abstract

**Einleitung:**

Die psychotherapeutische Sprechstunde am Arbeitsplatz (PT-A) bietet eine leicht zugängliche, kurzfristige Unterstützung für Beschäftigte mit psychischer Belastung. Ziel dieser Studie war es, die Implementierung der PT‑A in Hinblick auf ihre Bekanntmachung, den Zugang und die Nutzung zu untersuchen.

**Methoden:**

Die Daten wurden im Rahmen der randomisiert kontrollierten Studie (RCT) „Frühe Intervention am Arbeitsplatz“ (friaa) erhoben. Quantitativ wurden 46 betriebliche Akteur:innen (BA) hinsichtlich verwendeter Bekanntmachungswege sowie förderlicher Faktoren der Nutzung der PT‑A befragt. Aus der Baseline-Erhebung der RCT wurden die Zugangswege der 550 teilnehmenden Beschäftigten verwendet. Qualitativ wurden 7 BA zur Erwartung an die PT‑A sowie 22 Beschäftigte der RCT zu den Erfahrungen des Zugangs und der Nutzung der PT‑A interviewt.

**Ergebnisse:**

Die BA erhoffen sich von der PT‑A Wirkungen auf allen Ebenen der Prävention. Die meisten Betriebe machten die PT‑A über zentrale betriebsinterne Wege (z. B. Flyer, Intranet) oder mithilfe von individuellen Gesprächen (z. B. Sozialberatung, Betriebsärzt:innen) bekannt. Die Beschäftigten wertschätzten bei der zentralen betriebsinternen Bekanntmachung die Möglichkeit zur anonymen Teilnahme. Vorteil des unterstützten Zugangs z. B. über Sozialberatung oder Betriebsärzt:innen war die Erreichbarkeit von Beschäftigten ohne Behandlungserfahrungen, aber mit hohem Leidensdruck.

**Diskussion:**

Aufgrund der Ergebnisse wird empfohlen, die PT‑A sowohl zentral bei allen Beschäftigten zu bewerben als auch betroffene Beschäftigte in persönlichen Gesprächen direkt auf die PT‑A aufmerksam zu machen. Dadurch können verschiedene Zielgruppen erreicht werden und die Vorteile der anonymen Teilnahme bewahrt werden.

## Abstract (en)

**Introduction:**

Psychotherapeutic consultation at work (PT-A) offers easily accessible, short-term support for employees experiencing psychological stress. The aim of the study was to evaluate aspects of the implementation regarding announcement, access, and use of the PT‑A.

**Methods:**

The study was conducted as part of a randomized controlled trial (RCT) called “Early Intervention in the Workplace”. Forty-six company actors answered a questionnaire about how the companies announced the PT‑A to their employees, the barriers in the announcement of the PT‑A, and the beneficial factors of using the PT‑A. The access routes of the 550 participating employees were used from the baseline data of the RCT. Seven company actors were qualitatively interviewed about their expectations of the PT‑A and 22 participants of the RCT were interviewed about their experiences accessing and using the PT‑A.

**Results:**

The company actors hoped that the PT‑A would have an impact on all levels of prevention. Most companies announced the PT‑A centrally (e.g., flyers and intranet) as well as through individual recommendations (e.g., through the company’s social counselling and occupational health professionals). Employees appreciated the opportunity to participate anonymously in the central announcement. Advantages of the supportive access were seen in the accessibility of employees without treatment experience, employees with high levels of suffering, and employees who have not yet recognized their own need for treatment.

**Discussion:**

The results suggest that it is useful to announce the PT‑A centrally to all employees but also to recommend it personally to affected employees. By using both methods, different PT‑A target groups can be reached and the advantages of anonymous participation are retained.

## Einleitung

Die psychotherapeutische Sprechstunde am Arbeitsplatz (PT-A) wurde konzipiert, um als ergänzendes Angebot zur psychotherapeutischen Regelversorgung eine niedrigschwellige und kurzfristige psychotherapeutische Unterstützung für Beschäftigte mit subklinischen oder ausgeprägten psychischen Beschwerden bereitzustellen [1]. Sie wird von Psychotherapeut:innen durchgeführt und kann neben einer klinischen und arbeitsbezogenen Diagnostik auch eine psychotherapeutische Prävention oder Kurzzeittherapie sowie eine Begleitung der Wiedereingliederung beinhalten [1]. Dabei können je nach Bedarf sowohl arbeitsbezogene als auch private Problemlagen thematisiert werden. Das Ziel der PT‑A ist es, Symptome zu reduzieren, die Arbeitsfähigkeit zu sichern, eine schnellere Rückkehr zur Arbeit zu erreichen bzw. das Risiko für eine Erwerbsminderung zu senken [1, 2].

Bisher zeigten Studien zur Evaluation der PT‑A bzw. der psychosomatischen Sprechstunde im Betrieb (PSIB) eine hohe Zufriedenheit vonseiten der Beschäftigten, die das Angebot in Anspruch nahmen, sowie vielversprechende Ergebnisse hinsichtlich einer Verbesserung der Symptomatik [3, 4]. Jedoch wird die PT‑A von nur ca. 1–2 % der Beschäftigten aus Betrieben, die eine PT‑A anbieten, auch tatsächlich genutzt [5]. Dieser Anteil erscheint gering im Hinblick darauf, dass beispielsweise nach Krankenkassendaten der AOK im Jahr 2018 die 12-Monats-Prävalenz für eine psychische Erkrankung bei 30 % in der erwerbstätigen Bevölkerung lag [6]. Dabei zeigen Geschlechterunterschiede, dass weibliche Beschäftigte deutlich häufiger von psychischen Erkrankungen betroffen sind [6]. Die geringe Nutzungsquote deutet auf Barrieren bei der Implementierung, der Bekanntmachung und dem Zugang zur PT‑A hin.

Bislang wurden nur wenige betriebliche Maßnahmen, die auf die psychische Gesundheit abzielen, hinsichtlich ihrer Implementierung erforscht. Erste Ergebnisse weisen darauf hin, dass u. a. die Bedarfsermittlung sowie strukturelle Faktoren wie ausreichende Verfügbarkeit von Ressourcen (Personal, Finanzen, Zeit) und Einbindung in die Organisationspolitik entscheidende Faktoren für eine erfolgreiche Implementierung darstellen [7, 8]. Darauf aufbauend wurden 2 Bedarfsanalysen durchgeführt, aus denen sich Handlungsempfehlungen für die Gestaltung einer PT‑A bezüglich Umfang, Inhalt, Ort und Zeit ableiten lassen [5, 9]. Jedoch liefern diese Studien keine Rückschlüsse darauf, welche Bekanntmachungs- und Zugangswege für die PT‑A sinnvoll sind.

Qualitative Ergebnisse einer früheren Studie zeigen, dass Bewusstsein, Wissen und Engagement betrieblicher Akteur:innen (BA) weitere Determinanten für die Implementierung präventiver psychosozialer Interventionen darstellen [7]. Insbesondere die Einstellung der Führungspersonen zu psychosozialen Themen sowie die Unternehmenskultur stellen weitere wichtige Faktoren dar [10–12]. Demnach nehmen Beschäftigte aus Unternehmen mit einer positiven Gesundheitskultur, die sich durch Förderung von Kommunikation und Behandlung psychischer Erkrankungen auszeichnet, mit einer erhöhten Wahrscheinlichkeit Behandlungsangebote wahr [10, 11]. Eine positive Einstellung der BA gegenüber der PT‑A könnte somit ebenfalls eine entscheidende Rolle für die Implementierung spielen.

Das Ziel der vorliegenden Studie war, die Implementierung der PT‑A weitergehend zu analysieren. Dafür wurde untersucht:

1. welche Erwartungen die Betriebe an die Implementierung einer PT‑A hatten,
2. welche Wege der Bekanntmachung gewählt und welche hinderlichen Faktoren dabei wahrgenommen wurden,
3. über welche Zugangswege die Beschäftigten teilnahmen, welche Vor- und Nachteile die unterschiedlichen Zugangswege aus Sicht der Beschäftigten hatten sowie
4. welche weiteren Faktoren die Nutzung der PT‑A begünstigten.

## Methoden

**Abb. 1:**
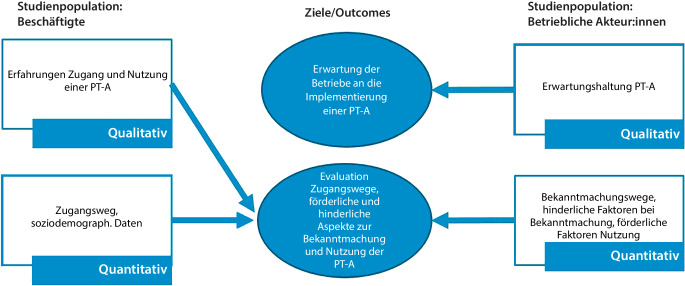
Verwendete Methodik aus qualitativer und quantitativer Erhebung (*PT‑A* psychotherapeutische Sprechstunde am Arbeitsplatz). (Quelle: eigene Abbildung)

Die Ergebnisse wurden im Rahmen der Studie „Frühe Intervention am Arbeitsplatz“ (friaa) gewonnen, einer multizentrischen randomisiert-kontrollierten Studie (RCT) zur Implementierung und Untersuchung der Wirksamkeit einer PT‑A in Deutschland [1]. Für die vorliegende Analyse wurden Teildaten der Erhebung zum ersten Messzeitpunkt (Baseline-Erhebung) der RCT, einer quantitativen Befragung der BA sowie einer qualitativen Prozessevaluation zusammengeführt (Abb. 1).

### Quantitative Erhebungen

#### Studiendesign und Studienpopulation

Es wurden Daten von allen teilnehmenden 550 Beschäftigten aus der Baseline-Erhebung dieser RCT verwendet. Die Teilnehmenden wurden über 60 kooperierende Betriebe und 11 überbetriebliche Multiplikator:innen (z. B. überbetriebliche betriebsärztliche Dienste und Sozialberatungen), aber auch über Werbeanzeigen auf sozialen Plattformen und in lokalen Zeitungen rekrutiert. Teilnehmen konnten Beschäftigte zwischen 18 und 65 Jahren mit einer Arbeitszeit von mind. 15 Wochenstunden, die subklinische oder klinische Symptome einer psychischen Erkrankung der sogenannten Common Mental Disorders aufwiesen (z. B. Angststörung). Weitere Informationen zu Studiendesign, Rekrutierung und Studienpopulation können dem Beitrag von Angerer et al. in diesem Themenheft und dem Studienprotokoll [1] entnommen werden.

Außerdem wurden die betrieblichen Ansprechpersonen von 54 kooperierenden Betrieben (z. B. Betriebsärzt:innen, Mitglieder des Betrieblichen Eingliederungsmanagements – BEM)^1^ und 11 Multiplikator:innen, die die Implementierung und die Vermittlung in die PT‑A unterstützten, zur Beantwortung eines Online-Fragebogens zur Implementierung der PT‑A eingeladen (im Folgenden BA genannt).

#### Messinstrumente

##### Bekanntmachungswege, hinderliche Faktoren bei der Bekanntmachung, förderliche Faktoren zur Inanspruchnahme der PT-A.

Die BA gaben im Fragebogen an (Mehrfachantworten möglich), wie sie Beschäftigte auf die Studie aufmerksam gemacht haben (14 Antwortmöglichkeiten), welche Schwierigkeiten sich bei den Bekanntmachungswegen zeigten (12 Antwortmöglichkeiten) und welche Faktoren es Beschäftigten erleichtert haben könnten, an der Studie teilzunehmen (15 Antwortmöglichkeiten). Aufgrund eines fehlenden Messinstruments, wurde ein solches in einem 3‑stufigen Verfahren entwickelt. Auf Basis bisheriger Veröffentlichungen zur Implementierung von betrieblichen Gesundheitsmaßnahmen wurden relevante Fragen und Antwortkategorien entwickelt [7, 8, 13]. Diese wurden von verantwortlichen Autor:innen der vorliegenden Studie ergänzt und von Studienmitarbeitenden aller Studienzentren der RCT begutachtet.

##### Zugangswege.

Im Rahmen der Baseline-Erhebung wurden durch die Studientherapeut:innen die Zugangswege zur Studie erhoben. Dabei wurde basierend auf einer Vorpublikation zur Gestaltung einer PT‑A [14] unterschieden zwischen *unterstützenden Zugangswegen*, bei denen Beschäftigte gezielt in persönlichen Gesprächen von Dritten (z. B. Betriebsärzt:innen, Vorgesetzten, Angehörigen) auf die RCT aufmerksam gemacht wurden, und *eigenständigen Zugangswegen*, bei denen Beschäftigte durch eine zentrale Bekanntmachung auf die RCT aufmerksam wurden (z. B. Flyer, Intranet, E‑Mail-Verteiler).

##### Berufsbezogene Informationen.

Die BA wurden nach ihrer beruflichen Position im Unternehmen und der von ihnen betreuten Betriebsgröße bzw. der Niederlassung gemäß der Definition der Europäischen Kommission für kleine und mittlere Betriebe gefragt [15]. Zusätzlich wurden 2 Ausprägungen für große Betriebe ergänzt (250–999 Beschäftigte und ≥ 1000 Beschäftigte). Die Größe der Betriebe, aus denen die teilnehmenden Beschäftigten stammen, wurde der Baseline-Erhebung entnommen.

##### Soziodemografische Informationen.

Die BA gaben die Geschlechterverteilung in dem Betrieb/der Niederlassung an, in dem/der die Beschäftigten auf die Studie aufmerksam gemacht wurden. Aus der Baseline-Erhebung wurden Angaben zum Alter und Geschlecht verwendet.

#### Quantitative Datenanalyse

Die Ergebnisse wurden deskriptiv mit Häufigkeiten und Mittelwerten dargestellt. Es wurden t‑Tests für unabhängige Stichproben zur Überprüfung von Altersunterschieden und *χ*^2^-Tests zur Überprüfung von Geschlechterunterschieden zwischen unterstützenden und eigenständigen Zugangswegen berechnet.

### Qualitative Erhebungen

Die qualitative Prozessevaluation der friaa-Studie basiert auf dem Verfahren der explorativ und gegenstandsangemessen ausgerichteten qualitativen Forschung. Die für die vorliegende Analyse genutzten Daten bilden Teilergebnisse der friaa-Prozessevaluation ab. Einbezogen wurden Ergebnisse aus 2 Fokusgruppen mit BA im Vorfeld der Implementierung der PT‑A sowie Daten aus Videointerviews mit Beschäftigten kurz nach Abschluss der Teilnahme an der PT‑A. Bei beiden Erhebungsformen, die per Video durchgeführt wurden, wurde eine Kombination aus narrativer und leitfadengestützter Befragung gewählt. Die Daten wurden extern per Audiogerät aufgezeichnet und transkribiert. Ausgewertet wurde für die vorliegende Studie inhaltsanalytisch nach Kuckartz und Rädiker [16]. Der Schwerpunkt lag auf der inhaltlich-strukturierenden Analyse jener Antworten, die sich auf das Erleben und Handeln in Hinblick auf die Implementierung der PT‑A bezogen. Ergebnisse bezüglich der Erwartungen an die PT‑A wurden aus Sicht der BA dargestellt. Aus den Beschäftigteninterviews wurden Ergebnisse zum Erleben des Zugangs zur PT‑A sowie förderliche Bedingungen dafür herausgearbeitet (Abb. 1).

## Ergebnisse

### Studienpopulation

An der quantitativen Befragung zur Implementierung der PT‑A nahmen 46 BA teil. Außerdem konnten für die quantitative Auswertung Daten der Baseline-Erhebung von allen 550 Beschäftigten, die an der RCT teilnahmen, genutzt werden. Von den Beschäftigten kamen etwa 80 % über kooperierende Betriebe und Multiplikator:innen und etwa 20 % von außerhalb dieser Betriebe in die Studie. An der qualitativen Befragung mittels Fokusgruppen nahmen 7 BA aus 5 Betrieben und 2 überbetrieblichen Diensten teil. Interviews wurden mit 22 Beschäftigten durchgeführt (Tab. 1).

**Tab. 1:** Beschreibung der Studienpopulation

	Quantitative Erhebung	Qualitative Erhebung
*Betriebliche Akteur:innen*		
Betriebe	40	5
Multiplikator:innen	6	2
*Berufsbezeichnung*		
Betriebsärzt:in	12	2
Betriebliche Sozialberater:in	4	2
BEM-Mitglied	13	–
Personalverwalter:in	6	–
BGM-Koordinator:in	15	–
Geschäftsführer:in	4	1
Personalleiter:in	4	2
Sonstige	4	–
*Betriebsgröße(n)*^*a*^		
1–9	0	–
10–49	4	–
50–249	6	–
250–999	9	1
≥ 1000	27	6
*Geschlechterverteilung der Beschäftigten im Betrieb, Median in % (Spanne; 8 fehlende Angaben der Betriebe)*		
Männlich	60 (15–87)	–
Weiblich	40 (13–85)	–
Divers	0 (0–2)	–
*Beschäftigte*		
Anzahl	550	22
Alter, Mittelwert (Standardabweichung)	46 (± 11)	47 (± 10)
*Geschlecht (%)*		
Männlich	247 (45)	9
Weiblich	301 (55)	13
Divers	1 (0)	–
*Betriebsgröße (2 fehlende Angaben)*		
1	1	–
2–9	10	–
10–49	38	–
50–249	60	2
250–999	100	2
≥ 1000	341	18

Angaben in absoluten Zahlen

*BEM* Betriebliches Eingliederungsmanagement, *BGM* Betriebliches Gesundheitsmanagement

^a^Klassifikation für Kleinstunternehmen, kleine und mittlere Unternehmen (KMU) der Europäischen Kommission [7] mit einer zusätzlichen Einteilung für Großbetriebe (250–999, ≥ 1000 Beschäftigte)

### Erwartungen betrieblicher Akteur:innen an die PT-A

**Abb. 2:**
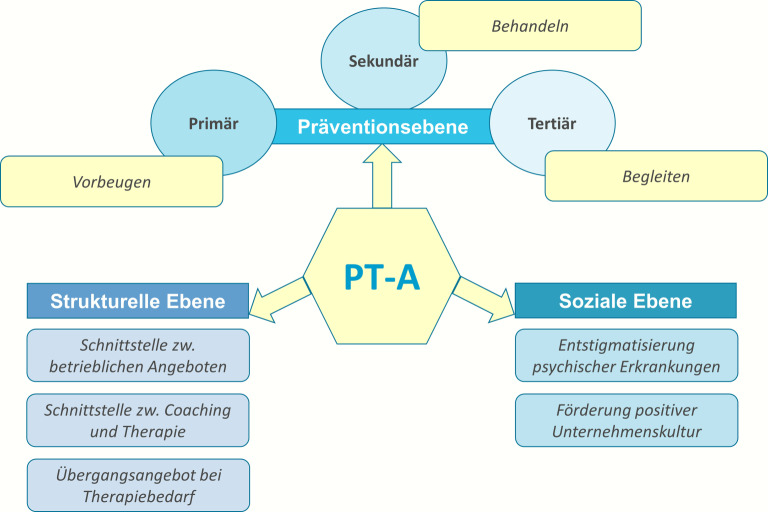
Erwartungen betrieblicher Akteur:innen an die psychotherapeutische Sprechstunde am Arbeitsplatz (PT-A). (Quelle: eigene Abbildung)

Die in den Fokusgruppen befragten BA erwarten von der PT‑A Wirkungen in 3 Bereichen: Prävention, strukturelle und soziale Ebene (Abb. 2). Dabei steht vor allem die Prävention (Primär‑, Sekundär- und Tertiärprävention) psychischer Erkrankungen im Mittelpunkt. Durch das niedrigschwellige Angebot soll frühzeitig auf therapeutischen Bedarf reagiert und die Verstärkung von Symptomen verhindert bzw. Symptome abgebaut werden, indem kritische Beanspruchungs- und Belastungssituationen erkannt und minimiert werden. Die schnelle therapeutische Intervention soll der Verhinderung von langen Arbeitsunfähigkeits(AU)-Zeiten dienen, wie zum Beispiel eine Betriebsärzt:in äußerte: „Ich denke auch, dass gerade dieser Ansatz, so früh wie möglich jemanden abzuholen, der optimalste Ansatz ist. Denn sobald sich was chronifiziert hat … wird es deutlich schwieriger.“ Zudem wird die PT‑A als professionelles Unterstützungsangebot bei der betrieblichen Wiedereingliederung eingeschätzt, das die nachhaltige Rückkehr an den Arbeitsplatz durch die Förderung der Passung individueller Bedarfe und betrieblicher Maßnahmen unterstützt. Führungskräfte bekommen mit der PT‑A ein „Handwerkszeug“, das sie Beschäftigten anbieten können, wenn sie eine Veränderung der Leistungsfähigkeit bemerken.

Auf der strukturellen Ebene wird die PT‑A durch ihren therapeutischen Ansatz als Mehrwert zwischen Coaching und Therapie und als Schnittstelle zwischen etablierten betrieblichen Angeboten und überbetrieblichem Gesundheitssystem verstanden. So äußert z. B. ein Mitarbeiter der Sozialberatung: „Ich finde dieses friaa-Angebot ist eine gute Entlastung für mich und ich glaub, da kann nochmal ganz andere Arbeit geleistet werden, als das, was ich hier tun kann.“ In Bezug auf die Unternehmenskultur zeigt die Etablierung einer PT‑A der Belegschaft nach Meinung der befragten BA, dass das Unternehmen psychische Erkrankungen ernst nimmt und sich um seine Beschäftigten „kümmert“. Die Implementierung der PT‑A wurde z. B. als Anlass genommen, überbetriebliche Kooperationen einzugehen und Veranstaltungen in den Unternehmen anzubieten, um das Wissen um psychische Erkrankungen zu erhöhen und die Entstigmatisierung zu fördern.

### Bekanntmachungswege

Die quantitative Befragung der BA ergab, dass bei dem unterstützenden Zugang zur PT‑A am häufigsten die Bekanntmachung durch Gespräche mit Betriebsärzt:innen (*n* = 27, 59 %), Vorgesetzen (*n* = 24, 52 %), Kolleg:innen (*n* = 21, 46 %) oder betrieblichen Interessensvertreter:innen (z. B. Betriebsrat; *n* = 22, 48 %) erfolgte. Es fanden aber auch Bekanntmachungen über Gespräche mit Personalabteilungen (*n* = 15, 33 %) und Sozialberatung (*n* = 15, 33 %) statt. Bei der zentralen Bekanntmachung verwendeten die meisten Betriebe Aushänge und Flyer (*n* = 28, 61 %) sowie das Intranet (*n* = 27, 59 %). Seltener wurde die PT‑A über E‑Mail-Verteiler (*n* = 17, 37 %), Firmen-Newsletter (*n* = 8, 17 %), Betriebsversammlungen (*n* = 6, 13 %), Mitarbeiterzeitschriften (*n* = 5, 11 %), betriebliche Gesundheitstage (*n* = 4, 9 %) oder Fortbildungen (*n* = 2, 4 %) bekannt gemacht.

#### Hinderliche Faktoren bei der Bekanntmachung

Die häufigsten genannten hinderlichen Faktoren bei Bekanntmachung der PT‑A waren die eingeschränkte Erreichbarkeit der Beschäftigten durch Homeoffice sowie die fehlende Möglichkeit, die PT‑A über Präsenzveranstaltungen bekannt zu machen (Tab. 2).

**Tab. 2:** Hinderliche Faktoren bei der Bekanntmachung der psychotherapeutischen Sprechstunde am Arbeitsplatz (PT-A) aus Perspektive der betrieblichen Akteur:innen

Hinderliche Faktoren bei der Bekanntmachung	Betriebe (*n* = 40)	Multiplikator:innen (*n* = 6)	Total
Erreichbarkeit der Beschäftigten durch Homeoffice eingeschränkt	10	1	11
Bekanntmachung war nicht über Präsenzveranstaltungen möglich	7	2	9
Zu wenig vorhandene betriebsinterne personelle Ressourcen (z. B. keine definierte Ansprechperson, kaum zeitliche Kapazität durch (andere) Arbeitsaufgaben)	6	n. a.	6
Die Integration des Angebotes in die Unternehmensprozesse war schwierig (z. B. keine Genehmigung einer universellen Bekanntmachung für die gesamte Belegschaft)	4	3	7
Die Bekanntmachung durch den/der Multiplikator:in erfolgte nicht hinreichend (bspw. wegen Zeitmangels)	n. a.	0	0
Unzureichende Verfügbarkeit von Informationsmaterial (z. B. gedruckte Flyer oder Plakate)	0	1	1
Es fand keine Kommunikation über das Angebot zwischen verschiedenen Hierarchieebenen statt	2	2	4
Es fand keine Kommunikation über das Angebot zwischen den Beschäftigten statt (Mundpropaganda)	6	1	7
Zu wenig Informationen vonseiten der friaa-Mitarbeitenden (z. B. zum Ablauf)	1	0	1
Die Bekanntmachung durch betriebliche Ansprechpersonen (z. B. Betriebsärzt:in) erfolgte nicht hinreichend (bspw. wegen Zeitmangels)	2	0	2
Erreichbarkeit der Beschäftigten durch Unternehmensstruktur (z. B. viele kleine Teams, Stabsstellen) schwierig/eingeschränkt	4	2	6
*Sonstige (Freitextantworten):*	*–*	0	*–*
Aufgrund der vielen Betriebsstätten innerhalb des Stadtgebietes konnten nicht überall Informationsmaterialien verteilt werden	1	–	1
Nicht alle Mitarbeitenden sind an das Intranet angebunden	1	–	1
Bekanntmachung durch Mailverkehr, Informationen im Intranet sowie Newsletter ohne Feedback der Mitarbeitenden	1	–	1
Es fand eine Online-Veranstaltung statt, bei der u. a. auf das Projekt aufmerksam gemacht wurde. Präsenz war in der Pandemie leider nicht möglich. Das hätte möglicherweise die Mundpropaganda besser gefördert	1	–	1

Angaben sind Häufigkeiten in absoluten Zahlen

*n.* *a.* nicht anwendbar (Frage wurde diesen Personen nicht gestellt)

### Zugang zur PT-A

**Abb. 3:**
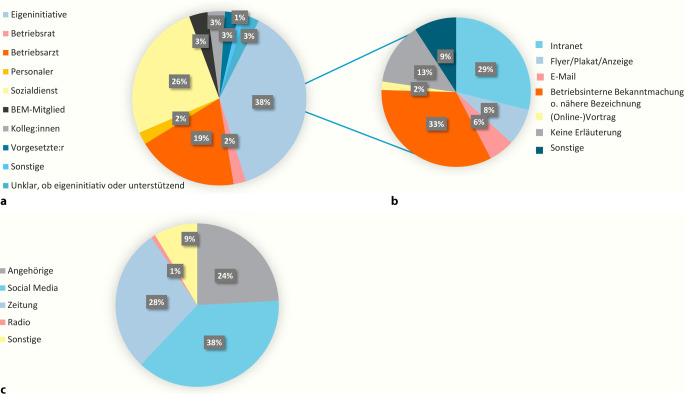
Zugangswege der Beschäftigten zur PT‑A (Anzahl = 550): **a** Zugang über die Betriebe (Anzahl = 434), **b** eigenständiger Zugang über die Betriebe (Anzahl = 163), **c** Zugang außerhalb der Betriebe (Anzahl = 116). *BEM* Betriebliches Eingliederungsmanagement, *PT‑A* psychotherapeutische Sprechstunde am Arbeitsplatz. (Quelle: eigene Abbildung)

Unter den 434 Beschäftigten, die sich über die Betriebe zur PT‑A anmeldeten, erfolgte der Zugang bei 38 % eigenständig (Abb. 3b). Von diesen erfuhren die meisten über das Intranet und andere nicht näher erläuterte betriebsinterne Bekanntmachungen von der PT‑A. Der Großteil der Beschäftigten, 58 %, meldete sich nach Gesprächen mit BA, wie z. B. dem Sozialdienst oder Betriebsärzt:innen (Abb. 3a). Dabei gab es keine Altersunterschiede zwischen Personen, die sich nach Gesprächen mit BA anmeldeten (M = 47, SD = 10), und Personen, die sich eigenständig meldeten (M = 45, SD = 11; t = −1,456; *p* = 0,15). Allerdings kamen unterproportional wenige Männer über einen eigenständigen (38 % männlich) und überproportional viele Männer über einen unterstützenden Zugang zur PT‑A (52 % männlich; 𝛘^2^ = 5,58; *p* = 0,018). Von den 116 Beschäftigten, die von außerhalb der kooperierenden Betriebe kamen, wurden die meisten über Social Media, durch Zeitungen oder durch Angehörige auf die PT‑A aufmerksam (Abb. 3c). Dabei gab es keine Altersunterschiede zwischen Personen, die sich aufgrund von Angehörigen (M = 42, SD = 12), und Personen, die sich eigenständig über Zeitungen, Radio oder Social Media meldeten (M = 43, SD = 12; t = 0,506; *p* = 0,614). Jedoch waren Beschäftigte, die sich aufgrund von Zeitungsartikeln meldeten, älter (M = 52, SD = 19) als Beschäftigte, die sich aufgrund von Social Media meldeten (M = 39, SD = 11; t = −5,853, *p* < 0,001). Auch hier kamen unterproportional wenige Männer über einen eigenständigen (30 % männlich) und überproportional viele Männer über einen unterstützenden Zugang durch Angehörige zur PT‑A (57 % männlich; 𝛘^2^ = 6,85; *p* = 0,009).

#### Zugang zur PT-A aus Sicht der Beschäftigten

##### Eigenständiger Zugang.

Anhand der qualitativen Interviews mit den Beschäftigten wird deutlich, dass jene Beschäftigten, die die PT‑A eigenständig aufgrund betriebsinterner Bekanntmachungen aufsuchten, vor allem schätzen, dass die Bekanntmachung alle Beschäftigten, einschließlich der Führungskräfte, adressierte. Dadurch wurde die PT‑A als sehr offenes und anonymes Angebot wahrgenommen. Als positiv wird empfunden, dass es eine eigenständige Entscheidung ist, wie mit dem Angebot umgegangen werden kann. Es wird kein Handlungsdruck erzeugt. Die Mehrheit der Befragten erwähnt positiv die Möglichkeit der „Bedenkzeit“. So äußert z. B. eine/r Beschäftigte: „… und da hat es dann aber nur ein paar Tage gedauert, wo dann für mich klar war, ja, warum eigentlich nicht.“ Diese Art des Umgangs mit der PT‑A erleben die Beschäftigten als niedrigschwellige Anregung, sich Hilfe zu suchen, wenn Belastung empfunden wird, ohne sich erklären zu müssen. Hervorgehoben werden zudem die unkomplizierte Ansprache sowie der Verweis auf das Thema psychische Gesundheit, der vermittelte, dass das Thema „für jeden interessant sein kann“, was zugleich zu einer Sensibilisierung führte.

##### Unterstützter Zugang.

Mittels der qualitativen Interviews wird deutlich, dass vor allem Beschäftigte, die (a) unter einem hohen Leidensdruck agieren und sich vor diesem Hintergrund Hilfe im betrieblich vorhandenen Gesundheitsangebot suchen und/oder (b) unerfahren mit therapeutischen Angeboten sind oder (c) ihr Problem nicht als therapeutisches Problem sehen, einen unterstützenden Zugang zur PT‑A suchten. Hilfreich ist dabei, wie z. B. ein Beschäftigter erzählt, „dass ich den Einstieg über die firmeninterne Sozialberatung gekriegt habe, dass ich direkt eine Empfehlung gekriegt habe und das hat mir die Hemmschwelle genommen“. Gleichzeitig verbinden die Beschäftigten mit dieser Empfehlung eine „Expertise“, nehmen die PT‑A damit als „seriös“ wahr und vertrauen dem Angebot dadurch stärker.

### Begünstigende Faktoren für die Nutzung der PT-A

#### Aus Perspektive der BA.

Bei der Befragung mittels Fragebogen wurden bei den förderlichen Faktoren von den BA am häufigsten die Aspekte „Anonymität des Angebotes“, „direkte Ansprechperson im Betrieb“ sowie „einfache Kontaktaufnahme zu den Studienmitarbeitenden“ angegeben (Tab. 3).

**Tab. 3:** Förderliche Faktoren für die Nutzung einer psychotherapeutischen Sprechstunde am Arbeitsplatz (PT-A) aus Perspektive der betrieblichen Akteur:innen

Förderliche Faktoren für die Nutzung	Betriebe (*n* = 40)	Multiplikator:innen (*n* = 6)	Total
Anonymität des Angebotes	24	5	29
Direkte Ansprechperson im Betrieb	19	1	20
Einfache Kontaktaufnahme zu den friaa-Mitarbeitenden	25	5	30
Kurze Wartezeiten bis zum Erstgespräch	19	3	22
Klare Struktur und Ablauf des Angebotes	9	1	10
Beratender Charakter des Angebotes	4	3	7
Positive Positionierung der Geschäftsführung zum Thema psychische Gesundheit und friaa	10	0	10
Akzeptierender Umgang der Beschäftigten zum Thema psychische Gesundheit im Betrieb	4	2	6
Kolleg:innen haben gute Erfahrungen mit friaa gemacht	8	0	8
Betriebsvorträge zum Thema „psychische Gesundheit“	4	1	5
Arbeitsplatzbezug des Angebotes wurde als sinnvoll betrachtet	3	2	5
Die Beschäftigten haben sich ausreichend informiert gefühlt über das Angebot	11	0	11
Die Beschäftigten haben gute Erfahrungen bei früheren betrieblichen Gesundheitsmaßnahmen gemacht	6	0	6
Angebot hat auf dem Betriebsgelände stattgefunden	3	0	3
*Sonstige (Freitextantworten):*			
Sehr freundliches Angebot und netter Kontakt zu den friaa-Kolleg:innen	1	–	1

Angaben sind Häufigkeiten in absoluten Zahlen

#### Aus Perspektive der Beschäftigten.

Die qualitativen Interviews mit den Teilnehmenden an der PT‑A bestätigen die quantitativen Ergebnisse. Vor allem die Anonymität des Angebots sowie der einfache und schnelle Zugang haben eine hohe Bedeutung für die Beschäftigten. Insgesamt war es den Befragten wichtig „zu wissen, worum es ging“, und dass sie sich informiert fühlten, was gleichfalls mit den Ergebnissen der Fragebogenbefragung korrespondiert. Auch diejenigen, die mit der PT‑A das erste Mal eine Therapie in Anspruch nahmen, erlebten den Zugang als unkompliziert. Bei allen Interviewpartner:innen war ein Leidensdruck vorhanden und es gab das Wissen, dass externe Therapeut:innen lange Wartezeiten haben, weshalb die PT‑A als ausgesprochen hilfreich bewertet wurde. Im Unterschied zur quantitativen Befragung der BA wurde durch fast alle interviewten Beschäftigten die Bedeutung des Arbeitsplatzbezugs im Rahmen der PT‑A betont. Außerdem spielten das Vertrauen in die Therapeut:innen als Menschen und das angenehme Erleben des Therapieorts als „nicht zu klinisch“ eine große Rolle.

## Diskussion

Die Ergebnisse zeigen, dass sich die Betriebe von der PT‑A Wirkungen auf allen Ebenen der Prävention erhofften und die PT‑A als ergänzendes Angebot zu bereits bestehenden betrieblichen Maßnahmen ansehen. Die Betriebe setzten sowohl auf zentrale, betriebsinterne Bekanntmachungswege als auch auf unterstützende Zugänge. Der größte Teil der Beschäftigten meldete sich über unterstützende Zugänge. Diese Zugänge erreichen insbesondere Beschäftigte mit hohem Leidensdruck, wenig Erfahrung mit psychotherapeutischer Behandlung oder fehlendem Bewusstsein für den Bedarf. Beim eigenständigen Zugang schätzten die Teilnehmer:innen die autonome Entscheidung und Berücksichtigung aller Beschäftigter bei der Bekanntmachung und Nutzung des Angebots. Die Anonymität und der schnelle Zugang zur PT‑A wurden sowohl von den BA als auch von Beschäftigten als förderliche Faktoren für die Nutzung angesehen.

Vorherige Studien zeigen, dass Betriebe sich von der PT‑A insbesondere eine Reduzierung der AU-Tage erhofften [2, 8] und dies für die PSIB auch in einer longitudinalen Studie nachgewiesen wurde [17]. Es wurden jedoch ebenfalls Bedenken geäußert, dass durch die PT‑A die AU-Tage ansteigen könnten, da alleine durch das Angebot der PT‑A eine Sensibilisierung für die Thematik und somit für den eigenen Bedarf entstehen könnte [2, 8]. Solche Bedenken wurden von den BA der vorliegenden Studie nicht geäußert. Die Erwartungshaltung der BA deckt sich jedoch mit weiteren Ergebnissen vorheriger Studien in Bezug darauf, dass eine Wirkung auf allen Ebenen der Prävention erhofft wird [2, 3]. Bezogen auf die Unternehmenskultur zeigten die BA, dass sie der PT‑A sehr positiv gegenüber eingestellt sind und den Beschäftigten signalisieren möchten, dass die psychische Gesundheit von Bedeutung ist. Vorherige Studien verdeutlichen, dass solch eine Unternehmenskultur mit einer höheren Wahrscheinlichkeit zum Aufsuchen von Behandlungen psychischer Störungen einhergehen kann [10, 11].

In der vorliegenden Studie wurden sowohl zentrale Bekanntmachungen als auch gezielte Ansprachen von Beschäftigten durch Betriebsärzt:innen und anderen BA genutzt. Eine zentrale Bekanntmachung wurde bereits in einer Studie zum *Employee Assistance Programme* (EAP) empfohlen, die zeigte, dass dies die Nutzungsquote erhöht [18]. Als positiver Aspekt für die zentrale Bekanntmachung wurde außerdem in einer Studie zur PSIB geäußert, dass so der Aspekt der Anonymität gewährleistet werden kann [2]. Da auch in dieser Studie die Anonymität der PT‑A als Vorteil vonseiten der BA und der Beschäftigten geäußert wurde, sollten zentrale Bekanntmachungswege bei zukünftigen Implementierungen berücksichtigt werden. Allerdings meldeten sich über eigenständige Zugänge unterproportional wenige männliche Beschäftigte an. Ergebnisse der Regelversorgung zeigen, dass Männer im Vergleich zu Frauen weniger dazu neigen, psychotherapeutische Behandlungen in Anspruch zu nehmen [19]. Daher könnte der unterstützende Zugang zusätzlich genutzt werden, um männliche Beschäftigte besser zu erreichen. Dies wird durch eine vorherige Studie bestätigt, die zeigte, dass der Zugang zur PSIB über die Betriebsambulanz zu einer Gleichverteilung von männlichen und weiblichen Teilnehmer:innen führte [20]. In vorherigen Studien wird häufig die Rolle der Betriebsärzt:innen in der Früherkennung psychischer Symptome und der Vermittlung der PT‑A beschrieben [3, 21, 22]. Da in dieser Studie der größte Anteil von Beschäftigten über betriebliche Sozialberatungen auf die PT‑A aufmerksam wurde, ergänzen die Ergebnisse, dass auch diese eine entscheidende Rolle bei der Implementierung einer PT‑A spielen. Weiterhin ergänzen die Ergebnisse, dass durch unterstützende Zugänge auch Personen erreicht werden, die den eigenen Bedarf einer psychotherapeutischen Behandlung bisher nicht wahrnahmen. Der selbst nicht wahrgenommene Bedarf ist laut Literatur einer der Hauptgründe, weswegen Personen mit Symptomen psychischer Störungen keine Behandlung aufsuchen [23–25]. Im Einklang mit einer früheren Empfehlung [2] sollte die PT‑A daher umfänglich beworben und sowohl zentrale als auch unterstützende Zugänge genutzt werden. Als hinderliche Faktoren für die Bekanntmachung gaben die BA die schwierige Erreichbarkeit der Beschäftigten im Homeoffice an. Eine andere Studie stellte dar, dass betriebliche Gesundheitsmaßnahmen schwieriger umzusetzen sind, wenn Beschäftigte an verschiedenen Standorten arbeiten [26]. Daher sollten solche Aspekte bei der Bekanntmachung berücksichtigt werden, um möglichst alle Beschäftigten zu erreichen.

Um Anzeichen psychischer Erkrankungen bei Beschäftigten frühzeitig zu erkennen und die Nutzung der PT‑A sowie weiterer psychosozialer Unterstützungsangebote zu empfehlen, wurden aufgrund früherer Studienergebnisse Schulungen für Führungskräfte empfohlen [8, 18]. In der vorliegenden Studie wurden von etwas über der Hälfte der Betriebe Gespräche durch Führungskräfte zur Bekanntmachung der PT‑A genutzt, aber nur ein Prozent der teilnehmenden Beschäftigten kamen aufgrund von Gesprächen mit Vorgesetzten. Dies könnte einerseits den Bedarf an Schulungen unterstreichen. Andererseits könnte dies aber auch auf mögliche Ängste vor Stigmatisierung zurückzuführen sein [10, 27, 28].

Hinsichtlich der Verfügbarkeit des Angebotes sollte in der PT‑A sichergestellt werden, dass der Zugang unkompliziert und kurzfristig möglich ist, da sie u. a. Wartezeiten für die psychotherapeutische Regelversorgung überbrücken soll [1]. Die Ergebnisse der vorliegenden Studie zeigen, dass dieser Aspekt erfolgreich in der PT‑A umgesetzt wurde, da sowohl BA als auch Beschäftigte die kurzen Wartezeiten besonders hervorhoben.

Die Ergebnisse zeigen außerdem, dass auch Bekanntmachungen außerhalb von Betrieben zu einer Teilnahme an der PT‑A führten. Sollen psychisch beanspruchte Beschäftigte unabhängig von der Betriebszugehörigkeit erreicht werden, z. B. bei einer Finanzierung über Krankenkassen, könnten Bekanntmachungswege unabhängig von Betrieben hilfreich sein. In der vorliegenden Studie haben sich insbesondere Bekanntmachungen über soziale Plattformen und Artikel in lokalen Zeitungen als kostengünstige und effiziente Möglichkeit gezeigt. Über soziale Plattformen wurden eher jüngere und über Zeitungsartikel eher ältere Beschäftigte erreicht.

### Einschränkungen

In der vorliegenden Studie lässt sich kein direkter Erfolg bestimmter Bekanntmachungswege ableiten, da nicht berechnet werden konnte, wie viele Beschäftigte durch die verschiedenen Wege tatsächlich von der PT‑A erfuhren. Außerdem ist eine Vermischung von zentralen Bekanntmachungen und unterstützenden Zugängen möglich, da Betriebe teilweise beides nutzten. So wurden einige Beschäftigte möglicherweise primär über eine zentrale Bekanntmachung auf die PT‑A aufmerksam, haben sich dann aber nach Gesprächen mit BA gemeldet.

Insgesamt waren die Nutzungsquoten in den Betrieben auch im Rahmen der RCT gering. Dies könnte zu einem Selektionsbias geführt haben, in dem z. B. Perspektiven von Beschäftigten mit einer positiven Einstellung zu betrieblichen Gesundheitsmaßnahmen überrepräsentiert sind. Zukünftige Studien sollten daher auch die Perspektive der Beschäftigten berücksichtigen, die die PT‑A nicht in Anspruch nehmen. Dies könnte weitere Hinweise auf förderliche und hinderliche Faktoren in den verschiedenen Zugangswegen und der Nutzung der PT‑A geben.

Nicht zuletzt werden in diesem Artikel nur Teilaspekte der Implementierung wie Bekanntmachung der Intervention und Erreichbarkeit der Beschäftigten thematisiert. Andere Implementierungsaspekte (z. B. Adhärenz, Kosten, Effektivität und Aufrechterhaltung nach dem RE-AIM-Modell [29]) werden nach Beendigung der Follow-up-Erhebungen der RCT separat veröffentlicht.

## Fazit

Basierend auf den Ergebnissen der vorliegenden Studie wird empfohlen, die PT‑A in den Betrieben sowohl zentral bei allen Beschäftigten als auch in individuellen Gesprächen durch Betriebsärzt:innen und Sozialberatung bekannt zu machen. Insbesondere durch unterstützende Gespräche können möglicherweise Personengruppen erreicht werden, die im Zusammenhang mit einer niedrigen Inanspruchnahme psychotherapeutischer Behandlungen stehen. Die Bekanntmachung der PT‑A außerhalb von Betrieben bietet eine zusätzliche Möglichkeit zur anonymen Teilnahme, wenn jene ohne Kooperation mit Betrieben angeboten werden sollte.

## Footnotes

1 Ansprechpersonen waren bei 6 der 60 kooperierenden Betriebe nicht mehr verfügbar und konnten daher nicht zur Befragung eingeladen werden.
